# A case of tamoxifen-induced hypertriglyceridemia monitoring the changes in lipoprotein fractions over time

**DOI:** 10.1186/s12902-021-00780-z

**Published:** 2021-06-09

**Authors:** Hayato Isobe, Masashi Shimoda, Yuki Kan, Fuminori Tatsumi, Yukino Katakura, Tomohiko Kimura, Atsushi Obata, Kenji Kohara, Shuhei Nakanishi, Tomoatsu Mune, Kohei Kaku, Hideaki Kaneto

**Affiliations:** 1 Division of Diabetes, Metabolism and Endocrinology, Kawasaki Medical School, 577 Matsushima, Kurashiki, 701-0192 Japan; 2 Professor with special assignment, Kawasaki Medical School, 577 Matsushima, Kurashiki, 701-0192 Japan

**Keywords:** Tamoxifen, Hypertriglyceridemia, Lipoprotein fraction, Type 2 diabetes

## Abstract

**Background:**

Tamoxifen, which is one of the selective estrogen receptor modulators (SERMs), can bring out life-threatening complication, e.g. hypertriglyceridemia-induced acute pancreatitis, although it is rare. We precisely report changes in lipoprotein metabolism before and after tamoxifen discontinuation because there have been few reports of it.

**Case presentation:**

47-year-old premenopausal woman with dyslipidemia, type 2 diabetes, nonalcoholic fatty liver disease and chronic kidney disease was prescribed tamoxifen as adjuvant therapy after operation of breast cancer. She experienced severe tamoxifen-induced hypertriglyceridemia several months after dosing tamoxifen. Before cessation of tamoxifen, lipoprotein fraction test revealed marked stagnation of VLDL and IDL metabolisms, resulting in severe hypertriglyceridemia (serum triglyceride level was 1881 mg/dL). Seven days after tamoxifen withdrawal, lipoprotein fraction test showed that the metabolisms of endogenous lipoproteins were changed drastically.

**Conclusions:**

From these results, we confirmed that tamoxifen certainly changes lipoprotein metabolism through suppression of post-heparin lipolytic activity. It is very important to evaluate the balance between benefit and risk before dosing tamoxifen and survey lipid profiles constantly during treatment to avoid life-threatening complication when prescription of tamoxifen is planned.

## Background

Tamoxifen is one of the selective estrogen receptor modulators (SERMs) widely prescribed as adjuvant therapy for breast cancer which is estrogen receptor positive, progesterone receptor positive, or both [1]. Approximately 70% of breast cancers are estrogen receptor positive [2]. Tamoxifen interferes with the estrogen-dependent proliferation of breast cancer cells [3] and reduces mortality and recurrence rate [4–6]. Although the adverse effects of tamoxifen are generally recognized to be mild, its use is associated with significantly increased risks of endometrial cancer, gastrointestinal cancers, strokes and pulmonary emboli [7]. In contrast, tamoxifen significantly decreased myocardial infarction deaths and was associated with a statistically insignificant decrease in myocardial infarction incidence [7]. Although several studies described small changes in plasma lipoprotein concentrations, most of the changes reduced the risk of cardiovascular disease [8–10]. On the other hand, tamoxifen can bring out life-threatening complication, e.g. hypertriglyceridemia-induced acute pancreatitis, although it is rare [11]. Therefore, consideration about the balance of potential benefits and risks is required when using tamoxifen for long term.

We experienced a case with marked hypertriglyceridemia after tamoxifen administration. In this case report, we precisely report the change of lipoprotein fractions and each apolipoprotein level before and after withdrawal of tamoxifen.

## Case presentation

A case is 47-year-old premenopausal woman with dyslipidemia, type 2 diabetes, nonalcoholic fatty liver disease and chronic kidney disease. In March 2016, she was diagnosed with diabetes and dyslipidemia in preoperative examination of uterine myoma at hospital of referral source. When she was referred to our hospital, she had significant disorder of glucose and lipid metabolism. The data of her blood test were as below; HbA1c 14.2%, plasma glucose 364 mg/dL, total cholesterol (TC) 315 mg/dL, triglyceride (TG) 698 mg/dL, LDL-cholesterol (LDL-C) 147 mg/dL and non HDL-cholesterol (non HDL-C) 261 mg/dL. We immediately started treatment for dyslipidemia with 2.5 mg of rosuvastatin, in addition to treatment for diabetes. In June of the same year, she was also prescribed 80 mg of fenofibrate to treat hypertriglyceridemia with fasting TG 579 mg/dL. Although levels of HbA1c and TG were improved to 6.4% and 247 mg/dL, respectively, fenofibrate was stopped because her renal function deteriorated to eGFR 43.3 ml/min/1.75m^2^ in September 2017. In March 2018, she was prescribed 400 mg of tocopherol nicotinate because the TG value was re-increased to 418 mg/dL, and TG level was continued in 200–300 mg/dL after dosing it. She was diagnosed as breast cancer in April of the same year and had the breast cancer operation in May and was prescribed 400 mg of tamoxifen as adjuvant therapy in August. In September of the same year, rosuvastatin was withdrawn by transient exacerbation of renal function due to dehydration. After then, serum TG concentration was continued in 200–270 mg/dL to December of the same year, but it was gradually worsened to 387 mg/dL in January and to 539 mg/dL in March 2019. She was hospitalized for treatment of hypertriglyceridemia in September 2019 because of the deterioration of TG level to 1881 mg/dL with asymptomatic increment of serum pancreatic amylase.

On admission, physical examination showed no goiter, no Cushing signs (e.g. central obesity, red striae cutis and buffalo hump etc.) and no obesity (BMI 22.6 kg/m^2^). No cutaneous and retinal signs of primary hypertriglyceridemia were detected. She had no alcohol abuse, but had an unbalanced diet with carbohydrates and no exercise habits. She also had no history of cardiovascular disease and acute pancreatitis. Although she had no family history of dyslipidemia or cardiovascular disease within the interview survey, we could not completely rule out hereditary disorders, such as familial combined hyperlipidemia, because we could not perform further investigation due to the patient’s special family situation. She was also prescribed 0.5 mg of glimepiride once daily and 0.75 mg of dulaglutide once weekly for type 2 diabetes, 400 mg of tocopherol nicotinate twice daily for dyslipidemia, 20 mg of tamoxifen as adjuvant therapy for breast cancer before admission.

**Fig. 1:**
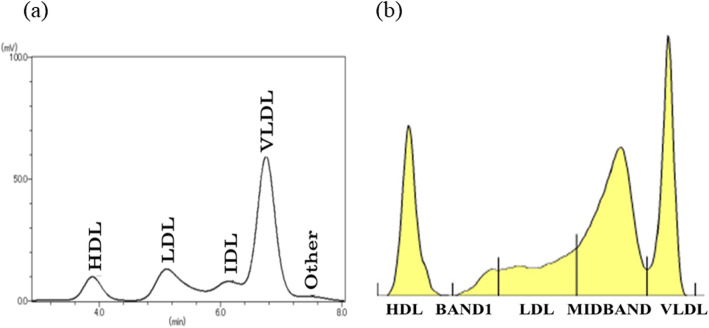
The results of lipoprotein fraction test in the next morning of admission. **a** Lipoprotein fraction test by using high performance liquid chromatography (HPLC) method and **b** polyacrylamide gel electrophoresis (PAGE) method in the next morning of admission

**Fig. 2:**
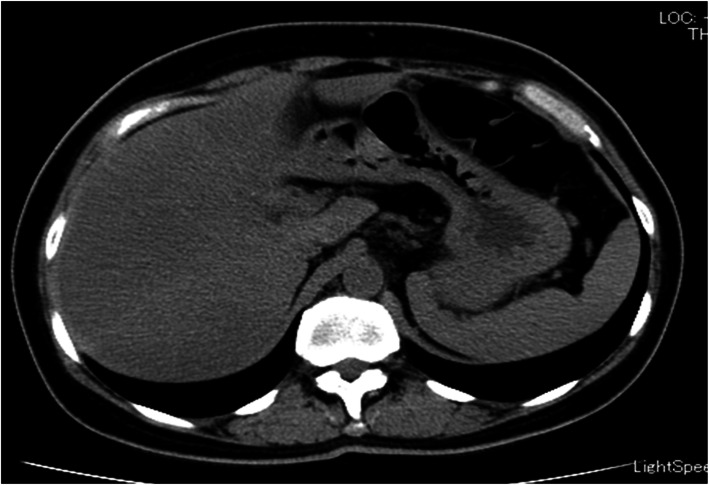
Abdominal computed tomography on admission. The image reveals the presence of fatty liver because the hepatic parenchymal density falls in patchy fashion

**Fig. 3:**
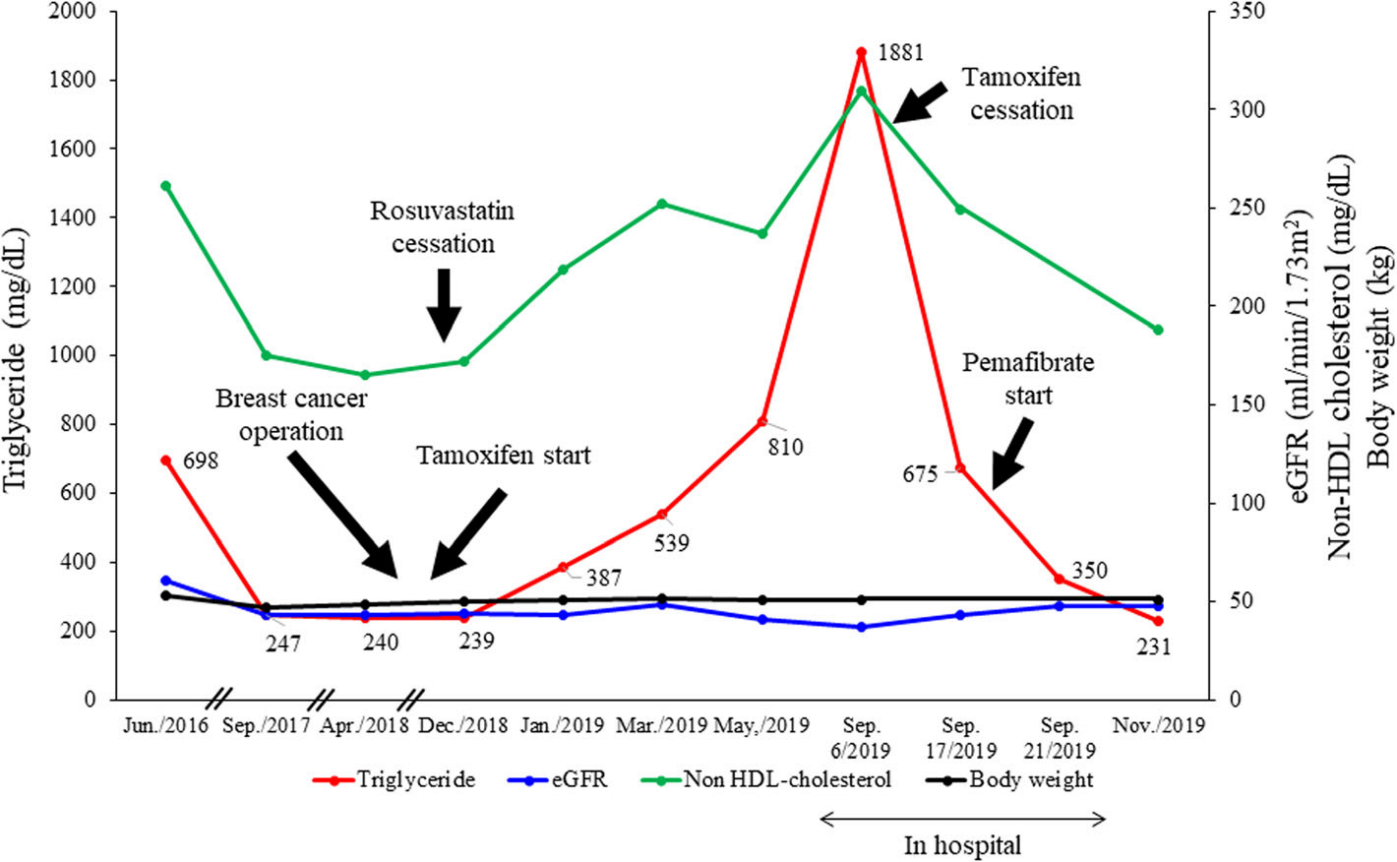
Clinical course of triglyceride, LDL-cholesterol, HbA1c and eGFR

She underwent fasting blood draws throughout the clinical course. The results in fasting blood sampling in the next morning of the admission were shown in Tables 1,2 and 3 and Fig. 1. As shown in Table 1 and Fig. 1, lipoprotein fraction test by using high performance liquid chromatography (HPLC) method indicated an increase of VLDL fraction mainly, and no chylomicron fraction. Serum standing test was also negative (data not shown), showing no presence of chylomicrons. This VLDL metabolic disorder was considered to be a factor in the marked increase of TG concentration. Remnant lipoprotein (RLP)-C and non HDL-C were also significantly augmented (Table 1). The degree of increase of apolipoprotein CIII level was higher than that of apolipoprotein CII level (Table 1), Furthermore, lipoprotein fraction test by using polyacrylamide gel electrophoresis (PAGE) method revealed the fraction which was named “BAND 1” with the smaller particle size than LDL, implying the existence of small dense LDL (Fig. 1b and Table 3). We also confirmed the existence of small dense LDL by calculating LDL-migration index (LDL-MI) and LDL-C/Apo B ratio (Table 3), the indicators of small dense LDL [12]. Furthermore, HbA1c level was 7.3%, and the image of abdominal computed tomography on admission revealed the presence of fatty liver because the hepatic parenchymal density fell in patchy fashion (Fig. 2), although the index of hepatic damage, such as ALT, AST, γGTP, was not increased (Table 2). Marked hypertriglyceridemia was thought to be due to multiple factors such as unbalanced diet, no exercise habit, uncontrolled diabetes, fatty liver, chronic kidney disease and tamoxifen therapy. On the other hand, there was little change in the clinical course before hospitalization of factors, such as eGFR, body weight (Fig. 3) and HbA1c, other than tamoxifen therapy. Therefore, we speculated that the main factor of rapid exacerbation of TG value was tamoxifen therapy.

**Table 1:** Lipid parameters in fasting blood sampling in the next morning of admission

Clinical parameters	Results	Units	Standard value
Parameters related with lipid metabolism			
TC	347	mg/dL	142–248
TG	964	mg/dL	30–149
HDL-C	34	mg/dL	40–103
LDL-C	68	mg/dL	65–139
non HDL-C	313	mg/dL	95–169
RLP-C	59.0	mg/dL	0.0–7.5
Prehepalin LPL mass	94	ng/mL	
Apo A-I	148	mg/dL	126–165
Apo B	158	mg/dL	66–101
Apo C-II	21.8	mg/dL	1.5–3.8
Apo C-III	63.4	mg/dL	5.4–9.0
Apo E	14.7	mg/dL	2.8–4.6
Lipoprotein fraction test (HPLC method)			
HDL	9.7	%	23.6–49.8
LDL	19.6	%	42.2–63.8
IDL	9.6	%	2.2–6.1
VLDL	59.6	%	2.6–13.9
Other	1.6	%	0.8–4.4
HDL (quantity)	33.6	mg/dL	40.6–91.4
LDL (quantity)	67.9	mg/dL	67.8–132.6
IDL (quantity)	33.4	mg/dL	3.8–12.5
VLDL (quantity)	206.8	mg/dL	4.9–22.8
Other (quantity)	5.4	mg/dL	1.5–9.1
Total cholesterol (quantity)	347	mg/dL	150–219

*TC* total cholesterol, *TG* triglyceride, *HDL-C* high density lipoprotein- cholesterol, *LDL-C* low density lipoprotein- cholesterol, *RLP-C* remnant lipoprotein- cholesterol, *LPL* lipoprotein lipase, *Apo* apolipoprotein, *HPLC* High Performance Liquid Chromatography

**Table 2:** Other parameters in fasting blood sampling in the next morning of admission

Clinical parameters	Results	Units	Standard value
Parameters related with glucose metabolism			
HbA1c	7.3	%	4.9–6.0
Plasma glucose	162	mg/dL	73–109
Parameters related with hepatic damage, renal function and pancreatic exocrine enzyme			
T-Bil	0.8	mg/dL	0.4–1.5
γGTP	37	U/L	9–32
ALT	15	U/L	7–23
AST	15	U/L	13–30
Cre	1.40	mg/dL	0.46–0.79
eGFR	32.9	ml/min/1.73m^2^	≥60
P-Amy	72	U/L	18–53
Elastase 1	314	ng/mL	0–300
Lipase	410	U/L	73–393
Urine qualitative analysis			
Uric protein	1+		–
Uric glucose	–		–
Uric ketone	–		–
Other parameters			
FT4	1.23	ng/mL	0.68–1.26
TSH	1.32	μIU/mL	0.75–4.12
Cortisol	22.6	μg/dL	4.5–21.1
ACTH	22.6	pg/mL	7.2–63.3
DHEA-S	170	μg/dL	19–231

*T-Bil* total bilirubin, *γGTP* γ-glutamyl transpeptidase, *ALT* alanine aminotransferase, *AST* aspartate aminotransferase, *Cre* creatinine, *eGFR* estimated glomerular filtration rate, *P-Amy* pancreatic amylase, *FT*; free thyroxine, *TSH* thyroid-stimulating hormone, *ACTH* adrenocorticotropic hormone, *DHEA-S* dehydroepiandrosterone sulfate

**Table 3:** Change of clinical parameters after admission

Parameters	Units	In hospital		After discharge
		Under dosing tamoxifen	7 days after tamoxifen withdrawal	2 months after dosing pemafibrate
Parameters related with lipid metabolism				
TC	mg/dL	341	282	234
TG	mg/dL	964	675	231
HDL-C	mg/dL	34	33	46
LDL-C	mg/dL	68	114	156
non-HDL-C	mg/dL	307	249	188
RLP-C	mg/dL	59.0	–	11.3
Apo A-I	mg/dL	148		153
Apo B	mg/dL	158	155	142
Apo C-II	mg/dL	21.8	14.5	9.5
Apo C-III	mg/dL	63.4	33.9	20.0
Apo C-III/C-II ratio		2.9	2.3	2.1
Apo E	mg/dL	14.7		5.5
LDL-C/ Apo B ratio		0.51	0.73	1.10
Lipoprotein fraction test (PAGE method)				
HDL	%	18	10	17
BAND 1	%	4	16	
LDL	%	16	12	44
MIDBAND	%	39	35	24
VLDL	%	23	27	15
LDL-MI		0.67	0.60	0.37
Other parameters				
HbA1c	%	7.3	–	7.0
Cre	mg/dL	1.40		1.00
eGFR	ml/min/1.75m^2^	32.9		47.5
P-Amy	U/L	72	53	–

*TC* total cholesterol, *TG* triglyceride, *HDL-C* high density lipoprotein- cholesterol, *LDL-C* low density lipoprotein- cholesterol, *RLP-C* remnant lipoprotein- cholesterol, *Apo* apolipoprotein, *PAGE* polyacrylamide gel electrophoresis, *LDL-MI* LDL-migration index, *Cre* creatinine, *eGFR* estimated glomerular filtration rate, *P-Amy* pancreatic amylase. LDL size was assessed by the migration of the LDL fraction, and LDL-MI was identified by the migration distance of LDL fraction relative to the HDL fraction

Before hospitalization, asymptotic increase of serum pancreatic amylase, elastase 1 and lipase was observed (Table 2). Although we considered hypertriglyceridemia-induced acute pancreatitis, we could not confirm any findings that positively suggested the onset of pancreatitis in abdominal computed tomography. Since the increase in exocrine pancreatic enzymes was relieved with the improvement of renal function, it was considered to be pseudo-hyperamylasemia (Table 3). Therefore, we did not instruct fasting during hospitalization.

**Fig. 4:**
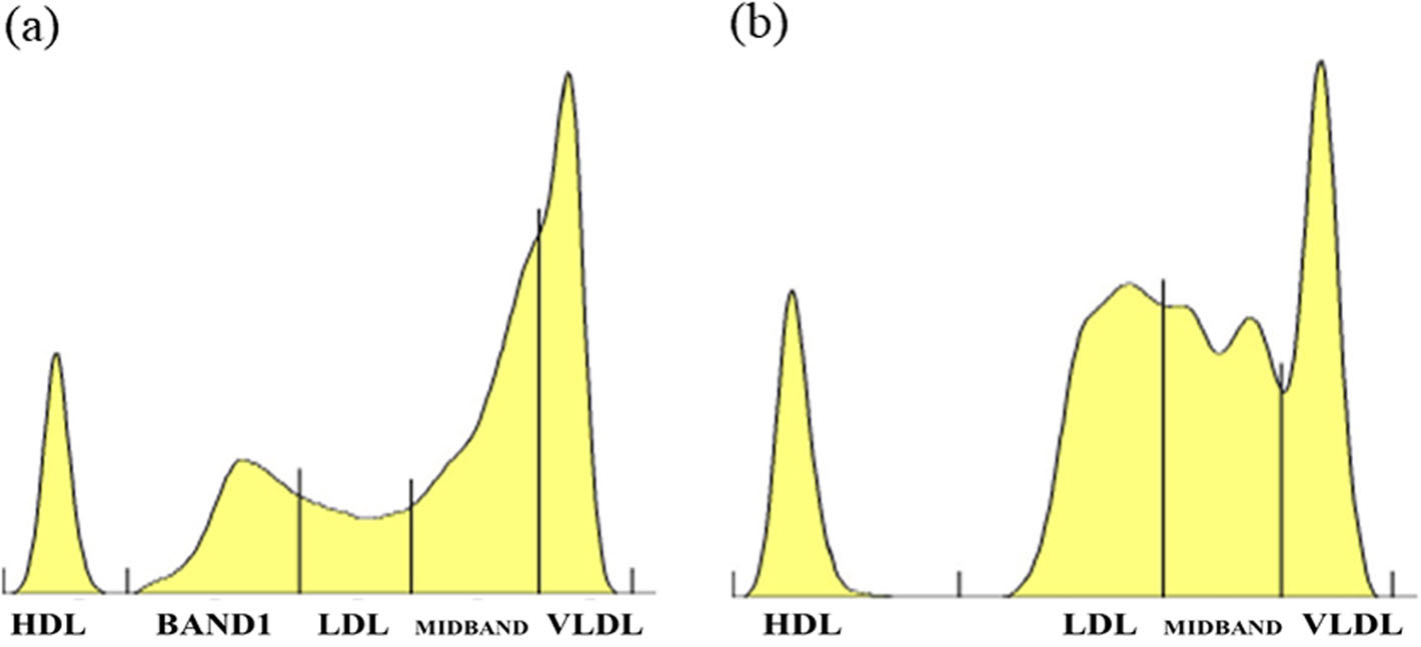
Change of lipoprotein fraction test by using PAGE method after admission. (**a**) Seven days after tamoxifen withdrawal and (**b**) two months after starting pemafibrate

After discontinuation of tamoxifen therapy, there were no conspicuous changes in the percentage of each fractions in lipoprotein fraction test by PAGE method seven days after tamoxifen withdrawal (Table 3), but the wave profile of each fractions changed prominently (Fig. 4a). These changes revealed that withdrawal of tamoxifen contributed to improvement of TG-rich lipoprotein metabolism, resulting in decrease of TG level. Furthermore, we prescribed pemafibrate, which is expected to have little effect on renal function, because improvement in renal function was confirmed after admission. After prescribing pemafibrate, the parameters, such as TG, non HDL-C and RLP-C, related with TG-rich lipoprotein metabolism were improved (Table 3), but LDL-C was increased (Table 3 and Fig. 4b). On the other hand, lipoprotein fraction test by PAGE method revealed the loss of small dense LDL fraction (Fig. 4b). LDL-MI and LDL-C/Apo B ratio were also changed from 0.67 to 0.37, 0.51 to 1.10, respectively (Table 3).

## Discussion and conclusions

We experienced an acute exacerbation of hypertriglyceridemia after tamoxifen administration in patient who had already suffered from hypertriglyceridemia due to chronic kidney disease (CKD), diabetes, nonalcoholic fatty liver disease (NAFLD).

unbalanced diet with carbohydrates and no exercise habit. Some authors have reported that severe hypertriglyceridemia by tamoxifen therapy usually occurs in those patients who have a previous diagnosis of familial hypertriglyceridemia or familial combined hyperlipidemia, and that in normolipidemic patients there is only moderate elevation of triglycerides [13]. Although she had no family history of dyslipidemia or cardiovascular disease within the interview survey, we could not completely rule out hereditary disorders, such as familial combined hyperlipidemia, because we could not perform further investigation due to the patient’s special family situation. The possible presence of hereditary dyslipidemia remains a factor in the marked exacerbation of hypertriglyceridemia with tamoxifen treatment. In this case, the onset of hypertriglyceridemia-induced acute pancreatitis due to tamoxifen therapy was not confirmed, but previous reports have revealed that tamoxifen increases serum triglyceride level and triggers hypertriglyceridemia-induced acute pancreatitis, whereas its incidence is rare [11]. Marked hypertriglyceridemia is an uncommon but well-established etiology of acute pancreatitis, with a reported incidence of 2–4% [14–16]. Data from European population studies reported that incidence of acute pancreatitis was 10–19% in subjects with severe hypertriglyceridemia, e.g. TG level is ≥1000 mg/dL [16]. Several studies have revealed that hypertriglyceridemia-induced acute pancreatitis is more likely to follow more severe clinical course, e.g. pancreatic necrosis, infected pancreatic necrosis, organ failure, prolonged hospitalization and death, compared to acute pancreatitis by other causes [17–19]. Therefore, it is particularly important to identify the cause in disturbance of lipid metabolism and treat hypertriglyceridemia in order to avoid the crisis of life.

Tamoxifen is one of SERMs with tissue-specific effects on estrogen signaling used predominantly for treatment and chemoprevention of breast cancers. SERMs have estrogen-like effects (agonistic action) on some tissues, but antiestrogen effects (antagonistic action) on other tissues [20]. In general, it is known that tamoxifen mainly affects lipid metabolism by its estrogenic actions, resulting in decreased LDL-C level and increased TG concentration [8–10]. Previous reports showed that estrogen impaired TG-rich lipoproteins metabolism and clearance due to suppression of post-heparin lipolytic activity [21–23]. Post-heparin lipolytic activity has been shown to consist of two activities: hepatic TG lipase (HTGL) and extrahepatic lipoprotein lipase (LPL). HTGL is the enzyme responsible for the hydrolysis of TG in different lipoproteins, contributing to the remodeling of VLDL remnants, as well as IDL, LDL and HDL. Furthermore, HTGL also acts as a ligand in accelerating the hepatic uptake of remnants and IDL particles [24]. On the other hands, LPL catalyzes the hydrolysis of TG in chylomicron and VLDL, producing chylomicron remnant and IDL, respectively. Furthermore, LPL can also act as a ligand for lipoprotein receptors to facilitate lipoprotein uptake [25]. Some groups reported that tamoxifen lowered activities of both LPL and HTGL, resulting in hypertriglyceridemia [26, 27]. The results of lipoprotein fraction test by using HPLC and PAGE methods on admission in our case also implied decreased activities of both LPL and HTGL (Fig. 1a and b). On the other hand, tamoxifen might have reduced the activity of LPL more than that of HTGL in our case, because the fractions of small dense LDL were detected clearly in the lipoprotein fraction test by PAGE method (Fig. 1b). Surprisingly, the metabolisms of endogenous lipoproteins were changed drastically 7 days after cessation of tamoxifen (Fig. 4a). Its change may imply that withdrawal of tamoxifen promptly alleviated the decrease in LPL activity. From these results, we confirmed that tamoxifen certainly changes lipoprotein metabolism through the effect on activity of LPL and HTGL.

In our case, the metabolism of TG-rich lipoproteins had already stagnated before tamoxifen administration although it is one of the causes that she could not have received the treatment with statin and/or fibrate because of renal side effect. Serum TG concentration gradually exacerbated some months after the start of adjuvant therapy for breast cancer using tamoxifen. In general, tamoxifen may need rather prolonged therapy to increase triglyceride level [13, 28], since short-term studies [29–31] failed to detect the changes in serum triglyceride levels. In the previous report [13], there are few cases of severe hypertriglyceridemia (TG > 1000 mg/dl) after dosing tamoxifen. The majority of these patients had past history of hypertriglyceridemia. In addition, when family history was provided, strong family history of dyslipidemia was evident. Additionally, tamoxifen is likely to increase TG level in patients with predisposition factors that may influence susceptibility to hypertriglyceridemia including increased TG concentration before prescription, such as diabetes, obesity, chronic kidney disease (CKD), nonalcoholic fatty liver disease (NAFLD), alcohol abuse, the concomitant use of certain medications and endogenous dyslipidemia (familial hypertriglyceridemia, familial combined hyperlipidemia) [32–34]. In our case, it did not take a long time to worsen TG concentration remarkably. It may be due to overlapping of above-mentioned various risk factors, e.g. uncontrolled TG, diabetes, CKD, NAFLD and hereditary dyslipidemia.

Lastly, we prescribed pemafibrate which is a novel selective peroxisome proliferator-activated receptor α modulator (SPPARMα) and has superior benefit-risk balance compared to conventional fibrates [35]. Pemafibrate resulted in further improvement in lipid metabolism as mentioned above in case presentation section.

In conclusion, we experienced a case with severe hypertriglyceridemia after administration of tamoxifen. Hypertriglyceridemia-induced acute pancreatitis might have brought out more severe clinical course. It is very important to evaluate the balance between benefit and risk before dosing tamoxifen and survey lipid profiles constantly during treatment to avoid life-threatening complication when prescription of tamoxifen is planned.

## Data Availability

Not applicable.

## Abbreviations

LDL: Low density lipoprotein
HDL: High density lipoprotein
eGFR: Estimated glomerular filtration rate
BMI: Body mass index
γGTP: γ-glutamyl transpeptidase
ALT: Alanine aminotransferase
AST: Aspartate aminotransferase
